# *In planta *assays involving epigenetically silenced genes reveal inhibition of cytosine methylation by genistein

**DOI:** 10.1186/1746-4811-8-10

**Published:** 2012-03-19

**Authors:** Sachiko Arase, Megumi Kasai, Akira Kanazawa

**Affiliations:** 1 Research Faculty of Agriculture, Hokkaido University, Sapporo 060-8589, Japan

**Keywords:** Cytosine methylation, Demethylating agents, Genistein, RNA-directed DNA methylation, Transcriptional gene silencing

## Abstract

**Background:**

Cytosine methylation is involved in epigenetic control of gene expression in a wide range of organisms. An increasing number of examples indicate that changing the frequency of cytosine methylation in the genome is a feasible tool to engineer novel traits in plants. Although demethylating effects of compounds have been analyzed in human cultured cells in terms of suppressing cancer, their effect in plant cells has not been analyzed extensively. Here, we developed *in planta *assay systems to detect inhibition of cytosine methylation using plants that contain a transgene transcriptionally silenced by an epigenetic mechanism.

**Results:**

Seeds of two transgenic plants were used: a petunia line that has been identified as a revertant of the co-suppression of the chalcone synthase-A (*CHS-A*) gene and contains *CHS-A *transgenes whose transcription is repressed; *Nicotiana benthamiana *plants that contain the green fluorescent protein (*GFP*) reporter gene whose transcription is repressed through virus-induced transcriptional gene silencing. Seeds of these plants were sown on a medium that contained a demethylating agent, either 5-azacytidine or trichostatin A, and the restoration of the transcriptionally active state of the transgene was detected in seedlings. Using these systems, we found that genistein, a major isoflavonoid compound, inhibits cytosine methylation, thus restoring transgene transcription. Genistein also restored the transcription of an epigenetically silenced endogenous gene in *Arabidopsis *plants.

**Conclusions:**

Our assay systems allowed us to assess the inhibition of cytosine methylation, in particular of maintenance of methylation, by compounds in plant cells. These results suggest a novel role of flavonoids in plant cells and that genistein is useful for modifying the epigenetic state of plant genomes.

## Background

Cytosine methylation is an epigenetic mark present in many eukaryotes, including plants, vertebrates and fungi [1], and plays an important role in various biological processes including regulation of gene expression, stability of the genome, cellular differentiation and development [2]. Transposons and repeats are frequently methylated in a wide range of species [3]. Loss of cytosine methylation induces reactivation and transposition of transposons [4-7], suggesting that cytosine methylation represents the primary mechanism of transposon suppression in host genomes [8]. Cytosine methylation also functions to maintain a repressed chromatin state and stably silence promoter activity [9]. A genome-wide analysis of *Arabidopsis thaliana *uncovered an interdependence between cytosine methylation and transcription [10].

Cytosine methylation in mammalian genomes occurs predominantly in the context of CG sequences. CG methylation is also the most common modification in plant genomes, but plant genomes also have cytosine methylation at CHG and CHH (where H is A, C or T) sequences. In plants, cytosine methylation can be established through RNA-directed DNA methylation (RdDM) [11], in which DOMAINS REARRANGED METHYLTRANSFERASE1 and 2 (DRM1 and DRM2), *Arabidopsis *orthologues of mammalian *de novo *methyltransferase DNMT3, are guided by small RNAs and targeted to DNA [12]. Whether all *de novo *cytosine methylation in *Arabidopsis *is guided by small RNAs is not known [12]. The maintenance of CG methylation requires METHYLTRANSFERASE1 (MET1), an orthologue of mammalian DNMT1, while that of non-CG methylation requires DRM1, DRM2 and the plant-specific methyltransferase CHROMOMETHYLASE3 (CMT3), which function redundantly [12]. On the other hand, a family of DNA glycosylases can remove cytosine methylation in plants [12].

Modification of the epigenetic state, in particular the frequency of cytosine methylation, has become a feasible tool to engineer novel traits in plants. Transcriptional gene silencing (TGS) has been induced by targeting small RNA to a transgene promoter via RdDM [11]. This approach has recently been applied to silence an endogenous gene using a viral vector, which led to the production of a plant that does not carry a transgene but has altered traits [13,14]. In addition to inducing changes via RdDM, changes in epigenetic state can also be induced randomly in the genome by inhibiting cytosine methylation. Although a method for targeted demethylation has not been developed for any organisms, transgenerational inheritance of a state of decreased methylation with an increased transcriptional activity has been observed for limited loci in plants [15]. These findings have prompted scientists to alter the cytosine methylation state of the genome that harbors novel epi-alleles. Examples of such attempts include a plant line that had a higher expression level of the *Xa21G *gene, which confers resistance against *Xanthomonas oryzae*, was produced in the progeny of rice plants treated with a demethylating agent [16]; various altered phenotypes were observed in a population of *Arabidopsis *recombinant inbred lines with epigenetically mosaic chromosomes consisting of wild-type and CG methylation-depleted segments that were derived from a cross between wild type and a *met1 *mutant [17]; exposure of *Arabidopsis *plants to various environmental stresses resulted in increased global genome methylation and a higher level of tolerance to stresses in the progeny [18]. Similarly, plants from an isogenic canola population, selected on the basis of respiration intensity, were used to generate sublines that have distinct characteristics and different epigenetic states [19].

One of the methods to modify the frequency of methylcytosine in the genome is to treat the organism with demethylating agents. 5-Azacytidine (5-azaC; trade name Vidaza) and its deoxy derivative 5-aza-2'-deoxycytidine (Decitabine; trade name Dacogen) were first synthesized over 40 years ago and are commonly applied inhibitors of cytosine methylation in plants and animals [20]. These reagents induce hypomethylation and reactivate transcriptionally repressed genes; hence, they have been used for cancer therapeutics to suppress hypermethylation of tumor suppressor genes in human. However, these toxic drugs are unstable in aqueous solution. More recently, 2-pyrimidine-1-β-D-riboside (Zebularine) induced the reactivation of hypermethylated genes in cultured human cells [21] and in plants [22]. Although this compound is stable in aqueous solutions, it raises considerable concerns about toxicity as a nucleoside analog. Besides nucleoside analogs, procainamide has been identified as an inhibitor of the human DNA methyltransferase DNMT1 [23]. Trichostatin A, known as a specific inhibitor of histone deacetylase, is also known to cause demethylation in *Neurospora crassa *[24] and plants [25,26] and downregulates DNA methyltransferase in human cells [27]. There is a growing list of cytosine methylation inhibitors in addition to these compounds (ref. [28]; and references therein).

In terms of finding compounds effective in suppressing cancer, natural products have been screened for inhibitory activity of cytosine methylation using human cancer cells. Through such analysis, plant secondary metabolites, polyphenols, were shown to be antagonists of cytosine methylation in cultured human cells. These compounds include (-)-epigallocatechin-3-*O*-gallate (EGCG) from green tea [29,30], genistein from soybean [30-32], caffeic acid and chlorogenic acid from coffee [33], polyphenols from Annurca apples [34], and lycopene from tomato [32]. *In vitro *studies revealed that some of these compounds inhibit DNA methyltransferase activity of human DNMT1 [31,33,35]. However, whether these compounds inhibit methylation in plant cells has not been examined.

In the present study, we focused on genistein, a major pharmacologically active isoflavone abundant in soybean [36] and examined whether genistein can function as a methylation inhibitor in plant cells. For that purpose, we developed two different *in planta *assay systems, both involving an epigenetically silenced transgene. In these systems, an increase in the mRNA level of the transgene, which is otherwise transcriptionally repressed, indicates inhibition of cytosine methylation. Through these assays, we demonstrated that genistein functions as a methylation inhibitor, thus identifying the first methylation inhibitor of plant origin that acts in plant cells.

## Results

### Detection of inhibition of cytosine methylation by genistein in transgenic petunia plants that have a transcriptionally silenced transgene

**Figure 1:**
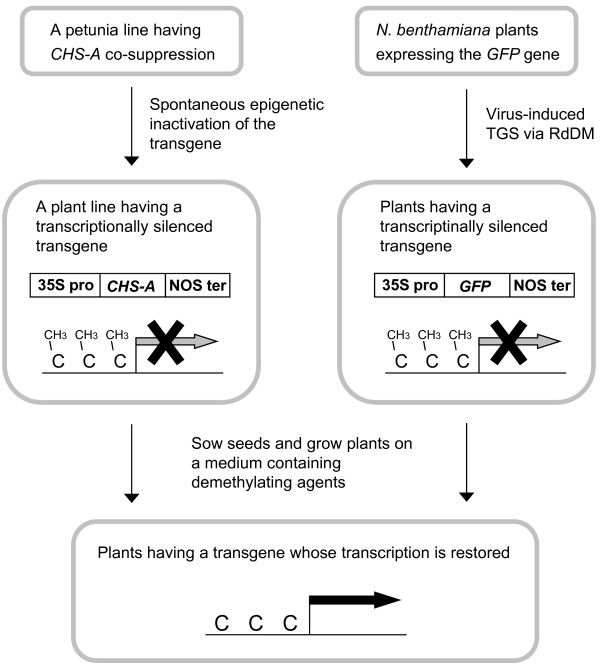
**Scheme of the system to detect inhibition of cytosine methylation**. Petunia plant line C002, which has transcriptionally silenced *CHS-A *transgenes, has been generated through spontaneous epigenetic changes involving cytosine methylation on CaMV 35S promoter (left; ref. [26]). *N*. *benthamiana *plants that have a transcriptionally silenced *GFP *gene were produced through virus-induced RNA-directed DNA methylation of the CaMV 35S promoter (right; ref. [37]). Seeds of these plants were sown on a medium that contained a potential demethylating agent. If the agent is inhibitory of cytosine methylation, the frequency of cytosine methylation in the promoters is reduced, resulting in the restoration of transcription from the promoters.

We have been maintaining plants of petunia C002 line that carry the chalcone synthase-A (*CHS-A*) transgene whose transcription is controlled by the *Cauliflower mosaic virus *(CaMV) 35S promoter. In this plant line, transcription of the *CHS-A *transgene is stably repressed through a spontaneous epigenetic change involving cytosine methylation of the CaMV 35S promoter [26]. We have shown previously that treatment of C002 plants with 5-azaC or TSA reduces cytosine methylation and restores transcription of the transgene. We came up with the idea of growing C002 plants on a medium that contained a potential demethylating agent and analyzed its effect based on restoration of transcriptional activity and reduction in the frequency of cytosine methylation in the transgene promoter. The scheme of this analytical system is shown in Figure 1 with the system using the green fluorescent protein (*GFP*) gene, which is described later.

**Figure 2:**
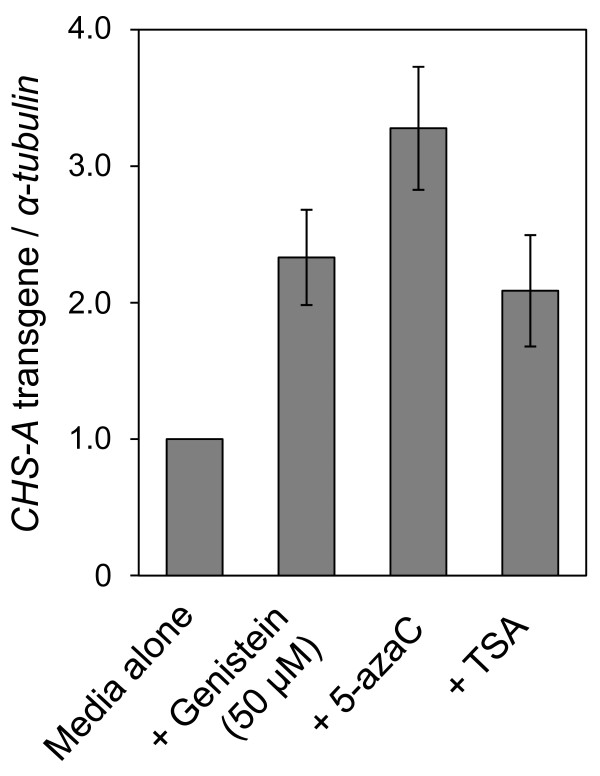
**Effects of genistein on reactivation of the transcriptionally silenced *CHS-A *transgene in petunia plants**. Real-time RT-PCR was conducted to analyze the mRNA levels of the *CHS-A *transgene in plants treated with genistein (50 μM), 5-azaC (20 μM) and TSA (6 μM). The mRNA levels were quantified relative to the mRNA level of *α-tubulin*. The value for control plants grown in a medium with no supplement ('Media alone') was set at 1. Data represent means and standard errors obtained from three replicates. Statistic analysis using Student's *t*-test indicated that the mRNA levels of *CHS-A *transgene in plants treated with these compounds were significantly higher than those of non-treated plants (*P *< 0.05).

**Figure 3:**
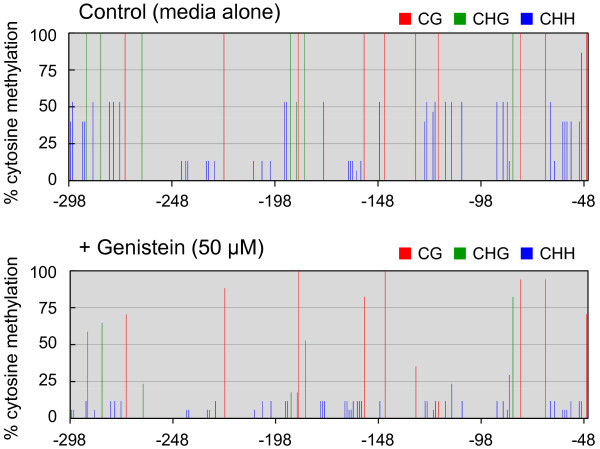
**Changes in frequency of methylcytosine in the CaMV 35S promoter as a consequence of treatment of C002 petunia plants with genistein**. Sequencing data for PCR products amplified from bisulfite-treated DNA have been compiled. The height of the vertical lines shows the frequency of methylcytosine at respective positions per total PCR clones sequenced. Red, green, and blue lines indicate frequencies of methylcytosine at CpG, CpHpG, and CpHpH sites, respectively. For control and genistein-treated plants, 15 and 16 clones were sequenced, respectively. Numbers below the line indicate nucleotide positions relative to transcription start site of the CaMV 35S promoter. The analyzed sequences cover the -298 to -47 region of the promoter.

Seeds of the C002 line were sown on a medium that contained genistein. For a positive control, plants were also grown in a medium supplemented with 5-azaC or TSA. RNA was isolated from bulked plants after growing for 1 month on the same plate, to analyze the level of mRNA transcribed from the *CHS-A *transgene using quantitative RT-PCR. The mRNA levels of the *CHS-A *transgene increased as a consequence of growing plants in the presence of genistein, indicating that the TGS is suppressed (Figure 2). In bisulfite sequencing analysis of cytosine methylation of the CaMV 35S promoter in plants treated with genistein, the methylation level was actually reduced (Figure 3). The frequency of methylcytosine was reduced in all sequence contexts, namely, CG, CHG and CHH (Additional file 1: Figure S1).

### Detecting inhibition of cytosine methylation by genistein in transgenic *Nicotiana benthamiana *plants that have a transcriptionally silenced transgene

We also analyzed the inhibitory effect of genistein on cytosine methylation using a different assay system (Figure 1, right). We previously developed a system that induces RNA-mediated heritable TGS using a virus vector [13,14,37]. In this system, a virus vector carrying a portion of a gene promoter efficiently induces cytosine methylation and TGS by targeting double-stranded RNA to the promoter, and the epigenetically silenced state of the gene is transmitted to subsequent generations. On the other hand, the virus that induced TGS is not transmitted to the next generation [13,37]. We used seeds produced on transgenic *N*. *benthamiana *plants, in which TGS of the *GFP *gene was systemically induced by infecting the plants with a recombinant virus that contained a portion of the CaMV 35S promoter, which controlled transcription of the *GFP *gene (ref. [37]; for details, see also Methods). Seeds produced on such plants were sown on a medium for germination, and the resultant plants were grown in the medium for 1 month.

**Figure 4:**
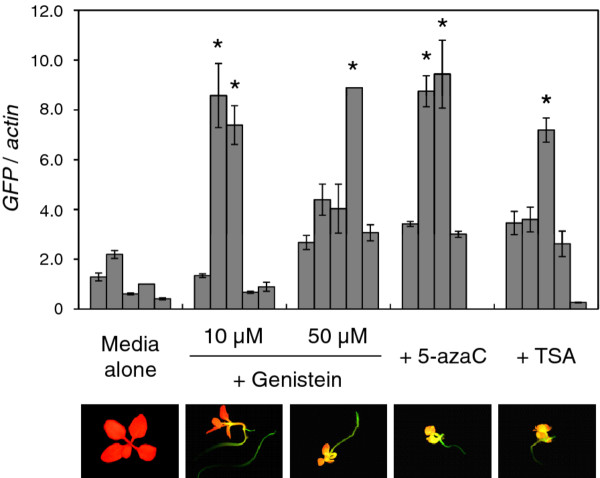
**Effects of genistein on reactivation of the transcriptionally silenced *GFP *gene in *N. benthamiana *plants**. Real-time RT-PCR was conducted to analyze the mRNA levels of the *GFP *gene in plants treated with genistein (10 μM or 50 μM), 5-azaC (20 μM) and TSA (2 μM). The mRNA levels were quantified relative to the mRNA level of the *actin *gene. Data for four or five individuals for each treatment are shown. The value for one of the control plants grown in a medium with no supplement ('Media alone') was set at 1. The data represent the means and standard errors obtained from three replicates. Statistic analysis was done using Bonferroni/Dunn test. Means that are indicated by asterisk are significantly different from those of all the five plants grown in a medium with no supplement (*P *< 0.05). A representative image of plants for each treatment is shown below the chart. Fluorescence was analyzed using a long-pass filter that allows detection of autofluorescence of chloroplasts colored in red.

Both GFP fluorescence and the mRNA level of the *GFP *gene indicated that the *GFP *gene expression was restored in plants grown in a medium that contained genistein as well as that contained 5-azaC or TSA (Figure 4). The advantage of this system is that GFP fluorescence allowed us to visually detect changes in the level of transgene expression in an individual plant. The extent of both growth inhibition and restoration of GFP fluorescence induced by each of these compounds differed between individual plants: the treated plant population comprised individuals that were highly affected and those that were not (for *GFP *mRNA level, see Figure 4; for GFP fluorescence, see Additional file 1: Figure S2). Such a difference among individual plants could be ascribed to the short half-life of these compounds in aqueous solution: e.g., the half-life of 5-azaC in mice is less than 6 h, and its residual effect lasts 1-2 days after administration [38]. If these compounds are not absorbed by proliferating cells of germinating plants while these compounds are active, then their full effect would not be realized. In addition, the low level of solubility of the compounds, especially genistein and TSA, in aqueous solution may also limit their uptake by plants. On the other hand, restoration of GFP fluorescence was not detected in any control plants grown in a medium with no supplement, which indicated that the effect was due to these compounds.

**Figure 5:**
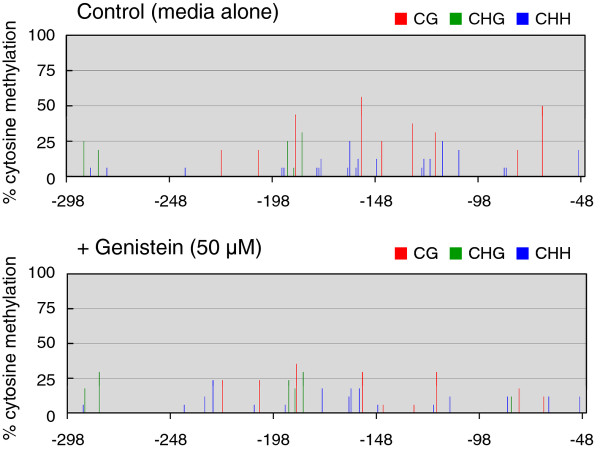
**Changes in frequency of methylcytosine in CaMV 35S promoter as a consequence of treatment of *N. benthamiana *plants with genistein**. For both control and genistein-treated plants, 17 clones were sequenced. Representative data of the analysis of DNAs isolated from a single control plant and three genistein-treated plants are shown. For other information, see the legend to Figure 3.

**Figure 6:**
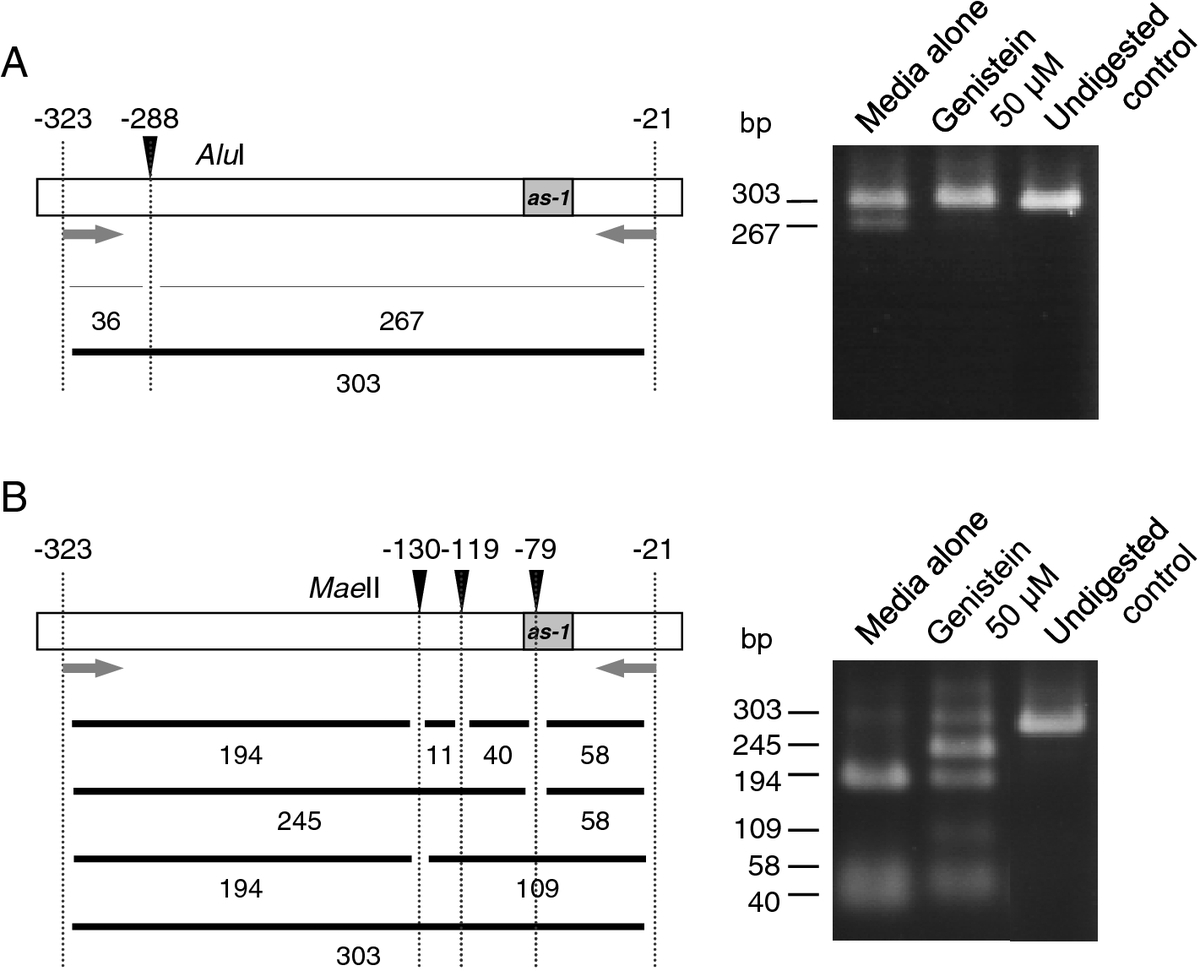
**Analysis of methylation status of CaMV 35S promoter in genistein-treated *N. benthamiana *plants by restriction digestion of DNA fragments amplified with PCR from bisulfite-treated DNA**. (A) Analysis of cytosine at position -288 (relative to the transcription initiation site) of the promoter using *Alu*I. (B) Analysis of cytosines at positions -130, -119, and -79 of the promoter using *Mae*II. Note that treatments of PCR-amplified fragments with *Alu*I and *Mae*II both resulted in lower levels of digestion when DNA isolated from genistein-treated plants was used for the analysis, indicating that genistein-treated plants have a lower frequency of cytosine methylation in the promoter. Sizes of DNA fragments (in bp) predicted by complete or partial digestions are indicated below the maps of the promoter. Arrows indicate primers for PCR. The position of the *cis*-acting *as-1 *element, to which binding of protein factor(s) is inhibited by cytosine methylation [60], is shown.

Bisulfite sequencing analysis showed that the frequency of cytosine methylation was reduced in the GFP-restored plants grown in a medium supplemented with genistein compared with plants grown in a medium with no supplement (Figure 5). Changes in the frequency of methylcytosine were also analyzed by digesting the DNA fragments amplified by PCR from bisulfite-treated DNA (Figure 6). In this experiment, tolerance of PCR products to digestion indicates lack of methylation of the cytosine at the restriction sites because of the conversion of cytosine by the bisulfite treatment. The results clearly indicated that genistein-treated plants had a lower frequency of methylation at the *Alu*I and *Mae*II sites in the CaMV 35S promoter than the control plants.

### Genistein reactivates a transcriptionally silenced endogenous gene in *Arabidopsis*

**Figure 7:**
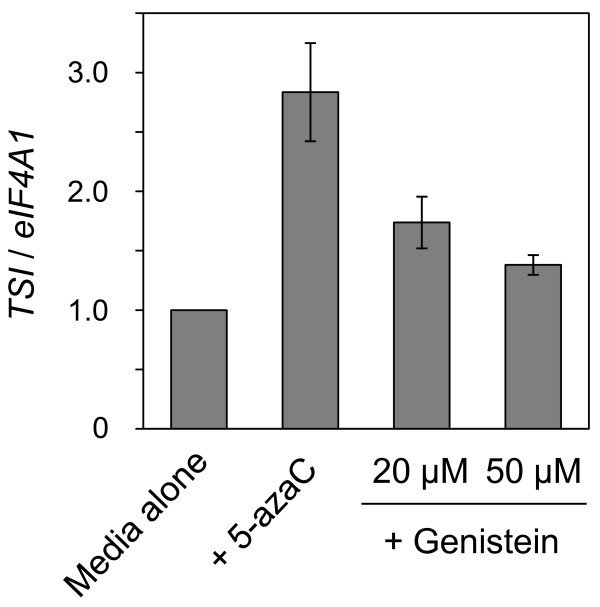
**Effects of genistein on mRNA level of a transcriptionally silenced endogenous gene in *Arabidopsis *plants**. Real-time RT-PCR was conducted to analyze mRNA level of *TSI *gene in *Arabidopsis *plants treated with genistein (20 μM or 50 μM) and 5-azaC (20 μM). The mRNA levels were quantified relative to the mRNA level of *eIF4A1*. The value of control plants grown in a medium with no supplement ('Media alone') was set at 1. Data represent the means and standard errors obtained from three replicates. Statistic analysis using Student's *t*-test indicated that the mRNA levels of *TSI *gene in plants treated with these compounds were significantly higher than those of non-treated plants (*P *< 0.05).

To determine whether genistein affects silenced state of an endogenous gene that is transcriptionally silenced, we analyzed *Arabidopsis *plants grown on medium supplemented with genistein. We analyzed changes in the mRNA level of *TRANSCRIPTIONALLY SILENT INFORMATION *(*TSI*), which is known to be silenced transcriptionally by an epigenetic mechanism involving cytosine methylation in vegetative tissues but reactivated by treatment with demethylating agents or in hypomethylation mutants [22,39]. In plants grown in medium that contained genistein, the mRNA level of *TSI *increased, although not as much as in 5-azaC-treated plants (Figure 7).

## Discussion

In the *in planta *assay systems that we have established, we can detect the restoration of the transcriptionally active state of a gene whose transcription is epigenetically repressed. Petunia C002 line arose as a spontaneous revertant plant from a *CHS-A *co-suppressed plant line in which transcripts of both the *CHS-A *transgene and endogenous *CHS-A *gene are degraded. Because transcription of the *CHS-A *transgene is repressed in C002 plants and small RNAs corresponding to both the *CHS-A *coding region and CaMV 35S promoter are not detected in this plant line [26], it is not likely that cytosine methylation of the CaMV 35S promoter is established in each generation of this plant line through *de novo *methylation. Such a lack of *de novo *methylation is unequivocally true of the promoter of the silenced *GFP *transgene in *N*. *benthamiana *plants. In this plant, the virus that induced cytosine methylation of the CaMV 35S promoter is eliminated during the meiosis and is not transmitted to the subsequent generation. As a consequence, the progeny plants that were used for the assay in the present study do not have viral RNAs that trigger *de novo *methylation on the CaMV 35S promoter [37]. Accordingly, reduction in the frequency of methylcytosine detected in the genistein-treated plants indicates inhibition of the process to maintain cytosine methylation rather than inhibition of *de novo *methylation. This notion is consistent with the fact that genistein inhibits the *in vitro *activity of human DNA methyltransferase DNMT1, an orthologue of *Arabidopsis *MET1 [31,32]. Thus, this assay system allows the detection of a compound's inhibitory activity that is specific to the maintenance process of the cytosine methylation. In this regard, our *in planta *assay systems can be used to dissect the functions of demethylating compounds. In addition, our assay systems may be useful for screening natural or artificial compounds that can inhibit cytosine methylation.

Using these assay systems, we found that genistein induced the reduction of the methylation of cytosines in all sequence contexts, namely, CG, CHG and CHH. Genistein may affect the catalytic function of more than one DNA methyltransferase that controls the maintenance of cytosine methylation. In *Arabidopsis*, in addition to controlling CG methylation, MET1 is required to maintain non-CG methylation: pre-existing CG methylation, or a chromatin mark associated with it, might be able to attract non-CG methylation [12]. Therefore, it is also possible that non-CG methylation is reduced as an indirect effect of inhibition of MET1 orthologue(s) by genistein. Alternatively, histone modification may be a target of genistein. Genetic studies have identified a link between histone and cytosine methylation: mutations in *CMT3 *and histone H3 lysine 9 (H3K9) methyltransferase gene *KYP *cause a reduction in CHG methylation (ref. [40]; and references therein). The CMT3 protein can interact with histone H3 when it is methylated at lysines 9 and 27, suggesting that these modifications recruit the CMT3 protein to target loci [40]. In human cancer cells, genistein can induce demethylation and acetylation of H3K9 [41]. Taking into account these observations, it is also conceivable that the observed reduction in cytosine methylation in plants treated with genistein is mediated by changes in histone modification, which result in failure to recruit DNA methyltransferase(s) to the targets. Such a link between cytosine methylation and histone modification is consistent with the restoration of transgene transcription in plants treated with TSA, an inhibitor of histone deacetylase. In fact, this compound can induce demethylation of cytosine in organisms including plants [24-26].

### Genistein may serve as an agent to modify the epigenetic state of a plant genome

In terms of producing plants with novel traits, changes in methylation levels can be induced by crossing plants with methyltransferase mutants. However, cytosine methylation affected by a mutation in a single methyltransferase gene is confined to a particular sequence context because a particular methyltransferase is responsible for methylation of cytosine in a specific sequence context. Moreover, mutants in cytosine methylation are available only in limited plant species. Therefore, genistein, like cytidine analogs such as 5-azaC or zebularine, has the potential advantage over the use of mutants in efficiently producing a novel epi-allele in plants. In addition, polyphenols such as genistein have a potential advantage over cytidine analogs. The mechanism of inhibition of methyltransferase by cytidine analogs involves incorporation of these compounds into DNA [42,43]. Incorporation of cytidine analogs results in a permanent alteration of the genome, often leading to mutation, whose process is also mediated by a DNA methyltransferase [44]. On the other hand, polyphenols are not incorporated into DNA and, hence, likely inhibit cytosine methylation without inducing mutation. This notion is consistent with the result of computational modeling, which suggests that EGCG directly interacts with the catalytic site of human DNMT1 to inhibit its activity [35]. Likewise, genistein might directly interact with MET1 orthologues and/or other methyltransferases without being incorporated into DNA in plants. Accordingly, in terms of inducing an epigenetic change rather than a mutation, the use of genistein may be preferred over nucleoside analogs, although the efficiency of the demethylation between genistein and nucleoside analogs may differ.

### Genistein inhibition of cytosine methylation suggests a novel role for flavonoids in plant cells

The reduction in the frequency of methylation suggests that exogenously applied genistein was active in the nucleus of the plant cell. Isoflavonoids are mostly present as glycosides or malonyl glycosides in plant tissues such as soybean seeds [45]. Whether these isoflavone glycoconjugates (e.g., genistin) or isoflavone malonyl glycoconjugates (e.g., malonylgenistin) affect cytosine demethylation the same as isoflavone aglycone (e.g., genistein) remains to be examined. Flavonoids, a major class of polyphenols, include flavonols, anthocyanins, proanthocyanidins (condensed tannins), and isoflavonoids. These compounds have diverse functions in plants, e.g., in flower pigmentation, UV protection, signaling, male fertility, and defense against pathogens [46-48].

Flavonoids are synthesized in the cytoplasm and are then deposited in a variety of cellular sites including the vacuole and cell wall or secreted to outside [47]. In addition to these locations, flavonoids can also be present in plant cell nuclei [49-52]. However, the significance of the accumulation of flavonoids in nuclei has not been clear, although anthocyanin and DNA have been presumed to form a protective complex against oxidative damage [53]. Here we found that genistein inhibited transcriptional repression not only of the transgene but also of an endogenous plant gene. The present results thus indicate the involvement of a flavonoid in the epigenetic control of transcription, a novel role for flavonoids in plant cells.

## Conclusions

We developed an *in planta *assay systems using plants of transgenic petunia and *N*. *benthamiana *that contain epigenetically silenced transgenes, which allow the detection of inhibition of cytosine methylation by a particular compound. Transcriptional repression of the *GFP *gene in *N*. *benthamiana *plants was induced by a recombinant virus, which is not transmitted to the subsequent generation. Therefore, the assay for restoration of *GFP *expression in the progeny plants in our system is particularly useful for detecting the inhibition of the maintenance process of cytosine methylation. Using these assay systems, we found that genistein has such activity. Genistein also had restoring effects on an epigenetically silenced endogenous gene in *Arabidopsis*, suggesting that the compound is useful for modifying epigenetic state of plant genomes.

## Methods

### Plant materials

Seeds of a transgenic petunia (*Petunia hybrida*) line C002 [26,54-56], designated CHS38P, were used. C002 is a line derived from a spontaneous revertant plant from the C001 line (designated as CHS38W), which was obtained by the transformation of wild-type plant V26 with the *CHS-A *transgene controlled by the CaMV 35S promoter and the NOS terminator [57]. The C001 line produces white flowers as a consequence of stable co-suppression of the *CHS-A *genes [56], while revertant line C002 produces pigmented wild-type flowers as a consequence of transcriptional repression of the *CHS-A *transgene, which occurred via an epigenetic mechanism involving cytosine methylation [26].

Seeds of transgenic *N*. *benthamiana *line 16c plants infected with a recombinant *Cucumber mosaic virus *containing a segment of the transgene promoter [37] were also used. The *N*. *benthamiana *16c line contains a single copy of the *GFP *transgene whose transcription is controlled by the CaMV 35S promoter [58]. Plants of this line were infected with the recombinant virus containing the -208 to -89 region (positions are relative to the transcriptional initiation site) of the promoter. We have found that the resultant plants systemically lose GFP fluorescence through the induction of TGS mediated by RdDM of the promoter, and the silenced state is stably transmitted to the subsequent generations [37]. In addition, seeds of *A*. *thaliana *ecotype Columbia (Col-0) were used for analyzing changes in the expression of endogenous genes.

### Treatment of plants with demethylating agents

Surface-sterilized seeds of *P*. *hybrida*, *N*. *bentahamiana *or *A*. *thaliana *were sown on plates of medium containing 1/2 concentration of MS salts [59] and 10 g/l sucrose. The pH of the medium was adjusted to 5.7 before autoclaving. Media were solidified with 0.8% (w/v) agar, amended with final concentrations of 20 μM aqueous 5-azaC, 2-6 μM TSA in methanol or 10-50 μM genistein in 80% methanol just before plates were poured. After 10 or 20 seeds were sown per plate, plates were incubated at a photoperiod of 16-h light/8-h dark, 26°C. Development of plants treated with these compounds was slightly affected (e.g., delay in germination). Each treatment was done in triplicate.

### Gene expression analysis

Isolation of total RNA and quantitative RT-PCR were done essentially as described previously [26]. The following primer pairs were used for the PCR: for the *CHS-A *transgene, CHS-trans + 12 F (5'-CTCATTTCTCTATTACTTCAGCC-3') and 2350 (5'-GTGCTTTGATCAACACAGTTTG-3'); for the petunia *α-tubulin *gene, tub1110F (5'-GCCACCATCAAGACCAAGC-3') and tub201R (5'-ACCTCAGCAACACTGGTTGA-3'); for the *GFP *gene, mGFP + 148 F (5'-ACTGGAAAACTACCTGTTCC-3') and mGFP + 344R (5'-TCAAACTTGACTTCAGCACG-3'); for the *N*. *benthamiana actin *gene, Nb-actF (5'-GAAGATACTCACAGAAAGAGG-3'); and Nb-actR2 (5'-GGAGCTAATGCAGTAATTTGG-3'); for the *Arabidopsis TSI *gene, TSI-F1 5'-TAGAGCAGTTAACCCGAACC-3') and TSI-R1 (5'-TAGCTTACTTCACCTAGAGTC-3'); for the *Arabidopsis *eukaryotic initiation factor 4A-1 (e*IF4A1*) gene (At3g13920), eIF4A-F1 (5'-CATGTTGAAGAGGCAGTCTC-3') and eIF4A-R1 (5'-GAAGAACACACCAACTTGGA-3'). Differences in mRNA level between plants were statistically analyzed using Student's *t*-test or Bonferroni/Dunn test. GFP fluorescence was examined with an epifluorescence microscope (MVX10; Olympus, Tokyo, Japan) equipped with a GFP mirror unit (U-MGFP HQ/XL and U-MGFP/XL; Olympus).

### Analysis of cytosine methylation

Cytosine methylation of the CaMV 35S promoter was analyzed by bisulfite sequencing. DNA was isolated from young seedlings grown on plates using Nucleon PhytoPure DNA extraction kit (Amersham Biosciences, Piscataway, NJ, USA). Bisulfite treatment of DNA, subsequent PCR amplification, and a control experiment to ensure the completion of the treatment were done as described previously [26].

## Competing interests

The authors declare that they have no competing interests.

## Authors' contributions

AK conceived and planned the study. SA, MK and AK carried out the experiments and drafted the manuscript. All authors read and approved the final manuscript.

## Supplementary Material

Additional file 1 **Figure S1**. **Summary of bisulfite sequencing analysis of CaMV 35S promoter in control and genistein-treated petunia C002 plants**. Red, green, and blue bars indicate frequencies of methylcytosine at CpG, CpHpG, and CpHpH sites, respectively. **Figure S2 GFP fluorescence of plants grown in a medium with no supplement and plants treated with genistein (10 μM or 50 μM)**, **5-azaC (20 μM)**, **and TSA (2 μM)**. Images of five *N*. *benthamiana *plants are shown for each treatment. Fluorescence was analyzed using a long-pass filter that allows detection of the red autofluorescence of chloroplasts. Click here for file
