# Erratum: *In planta* assays involving epigenetically silenced genes reveal inhibition of cytosine methylation by genistein

**DOI:** 10.1186/s13007-015-0058-6

**Published:** 2015-03-21

**Authors:** Sachiko Arase, Megumi Kasai, Akira Kanazawa

**Affiliations:** Research Faculty of Agriculture, Hokkaido University, 060-8589 Sapporo, Japan

## Erratum

The legend of Figure 6 in our original article [1] is changed to the following description because of an error in the identity of samples:

In accordance with this change, descriptions relevant to this figure: “Changes in the frequency of methylcytosine were also analyzed by digesting the DNA fragments amplified by PCR from bisulfite-treated DNA (Figure six (Figure 1 here)). In this experiment, tolerance of PCR products to digestion indicates lack of methylation of the cytosine at the restriction sites because of the conversion of cytosine by the bisulfite treatment. The results clearly indicated that genistein-treated plants had a lower frequency of methylation at the *Alu*I and *Mae*II sites in the CaMV 35S promoter than the control plants.” should be moved from page 4, line 48 of the right column, to page 4, line 12 of the left column.

**Figure 1:**
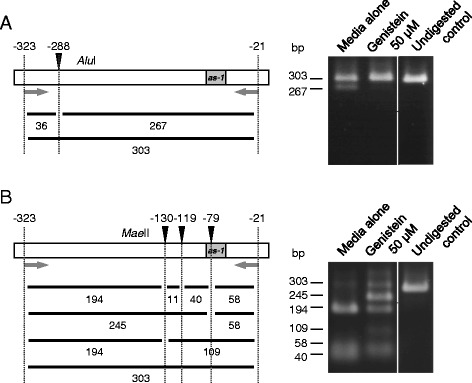
**Analysis of methylation status of CaMV 35S promoter in genistein-treated C002 petunia plants by restriction digestion of DNA fragments amplified with PCR from bisulfite-treated DNA. (A)** Analysis of cytosine at position –288 (relative to the transcription initiation site) of the promoter using *Alu*I. **(B)** Analysis of cytosines at positions –130, –119, and –79 of the promoter using *Mae*II. Note that treatments of PCR-amplified fragments with *Alu*I and *Mae*II both resulted in lower levels of digestion when DNA isolated from genistein-treated plants was used for the analysis, indicating that genistein-treated plants have a lower frequency of cytosine methylation in the promoter. Sizes of DNA fragments (in bp) predicted by complete or partial digestions are indicated below the maps of the promoter. Arrows indicate primers for PCR. The position of the *cis*-acting *as-1* element, to which binding of protein factor(s) is inhibited by cytosine methylation [60], is shown. The profiles of DNA samples that were not adjacent to each other in the original gel were separated by a line.

The corrected Figure six (Figure 1 here), in which the profiles of DNA samples that were not adjacent to each other in the original gel are separated by a line, is shown here.
