# Supplementary material for: In planta assays involving epigenetically silenced genes reveal inhibition of cytosine methylation by genistein
Source: Plant Methods. 2012 Mar 19;8:10. doi: 10.1186/1746-4811-8-10 (PMC3362751; doi:10.1186/1746-4811-8-10)

Additional files

Figure legends

Additional file Figure 1 Summary of bisulfite sequencing analysis of CaMV 35S promoter in control and genistein-treated petunia C002 plants. Red, green, and blue bars indicate frequencies of methylcytosine at CpG, CpHpG, and CpHpH sites, respectively.

Additional file Figure 2 GFP fluorescence of plants grown in a medium with no supplement and plants treated with genistein (10 µM or 50 µM), 5-azaC (20 µM), and TSA (2 µM). Images of five *N. benthamiana* plants are shown for each treatment. Fluorescence was analyzed using a long-pass filter that allows detection of the red autofluorescence of chloroplasts.

Additional file Figure 1

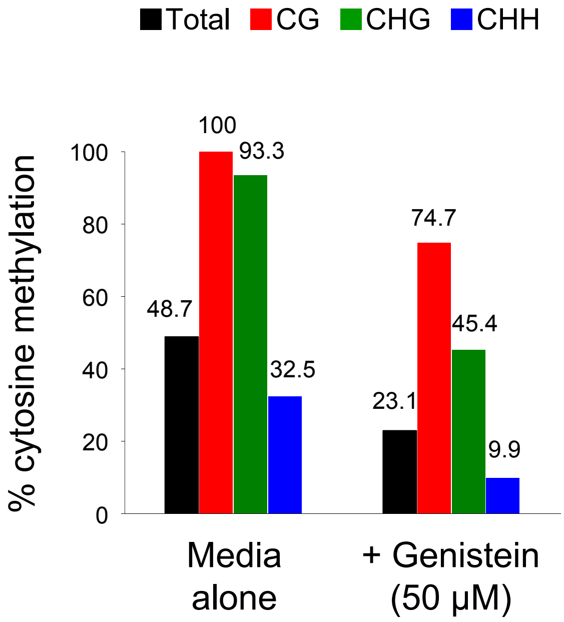

Additional file Figure 2

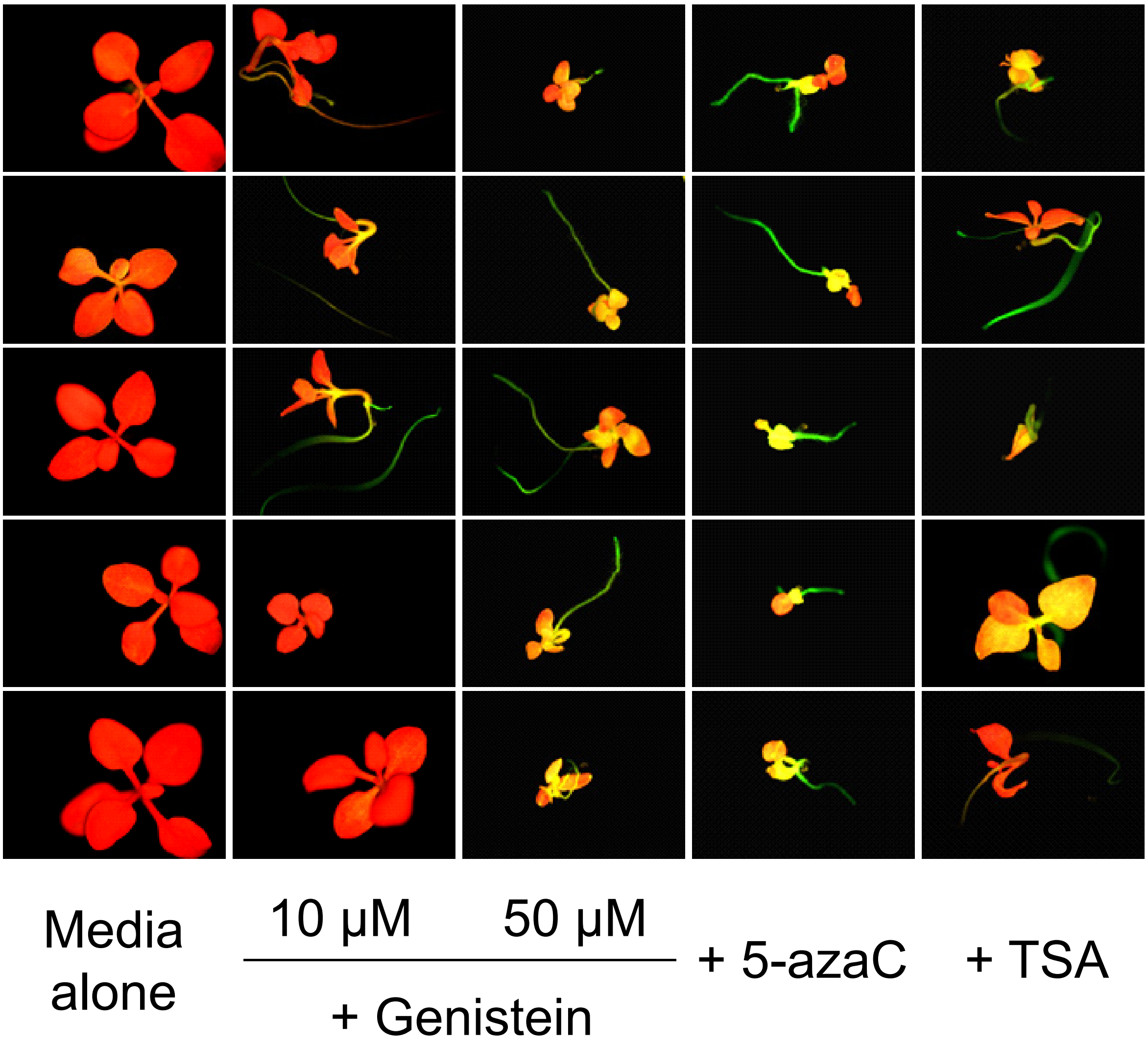

Supplement: Additional file 1 — Figure S1. Summary of bisulfite sequencing analysis of CaMV 35S promoter in control and genistein-treated petunia C002 plants. Red, green, and blue bars indicate frequencies of methylcytosine at CpG, CpHpG, and CpHpH sites, respectively. Figure S2 GFP fluorescence of plants grown in a medium with no supplement and plants treated with genistein (10 μM or 50 μM), 5-azaC (20 μM), and TSA (2 μM). Images of five N. benthamiana plants are shown for each treatment. Fluorescence was analyzed using a long-pass filter that allows detection of the red autofluorescence of chloroplasts. [file 1746-4811-8-10-S1.PDF]
